# Studies on the aerobic utilization of synthesis gas (syngas) by wild type and recombinant strains of *Ralstonia eutropha* H16

**DOI:** 10.1111/1751-7915.12873

**Published:** 2017-10-13

**Authors:** Daniel Heinrich, Matthias Raberg, Alexander Steinbüchel

**Affiliations:** ^1^ Institut für Molekulare Mikrobiologie und Biotechnologie Westfälische Wilhelms‐Universität Münster Germany; ^2^ Environmental Sciences Department King Abdulaziz University Jeddah Saudi Arabia

## Abstract

The biotechnical platform strain *Ralstonia eutropha* H16 was genetically engineered to express a *cox* subcluster of the carboxydotrophic *Oligotropha carboxidovorans* OM5*,* including (i) the structural genes *coxM*, ‐*S* and ‐*L*, coding for an aerobic carbon monoxide dehydrogenase (CODH) and (ii) the genes cox*D*, ‐*E*, ‐*F* and ‐*G*, essential for the maturation of CODH. The *cox*_*Oc*_ genes expressed under control of the CO_2_‐inducible promoter P_*L*_ enabled *R. eutropha* to oxidize CO to CO_2_ for the use as carbon source, as demonstrated by ^13^ CO experiments, but the recombinant strains remained dependent on H_2_ as external energy supply. Therefore, a synthetic metabolism, which could be described as ‘carboxyhydrogenotrophic’, was established in *R. eutropha*. With this extension of the bacterium's substrate range, growth in CO‐, H_2_‐ and CO_2_‐containing artificial synthesis gas atmosphere was enhanced, and poly(3‐hydroxybutyrate) synthesis was increased by more than 20%.

## Introduction

The β‐proteobacterium *Ralstonia eutropha* H16, currently named as *Cupriavidus necator* H16, represents a model organism for autotrophic lifestyle and has been studied extensively for its efficient utilization of carbon dioxide and hydrogen as carbon and energy sources (Friedrich and Schwartz, 1993). By means of enzymes of the Calvin‐Benson‐Bassham (CBB) cycle, *R. eutropha* H16 fixes CO_2_ from the atmosphere as the sole source of carbon as well as from CO_2_, generated from its own intracellular oxidation of organic carbon compounds like formate (Kärst and Friedrich, 1984; Shimizu *et al*., 2015). When growing lithotrophically, *R. eutropha* H16 oxidizes molecular hydrogen by a soluble and a membrane‐bound hydrogenase, which are tolerant to oxygen, as well as to carbon monoxide (Buhrke *et al*., 2005; Burgdorf *et al*., 2005; Bürstel *et al*., 2016).

Several approaches have been made to exploit *R. eutropha's* efficient chemolithoautotrophic lifestyle to potentially capture CO_2_ from waste‐ or industrial exhaust gases for the conversion to various products of increased value. These so‐called second‐generation bioproducts include polymers, fuels and fine chemicals (Cook and Schlegel, 1978; Tanaka and Ishizaki, 1994; Müller *et al*., 2013; Lu and Yu, 2017). An alternative form of a gaseous feedstock that can be produced from fossil as well as from renewable resources, is the platform chemical synthesis gas (syngas), which has gained considerable attention for the use as cheap, energy‐rich and abundant feedstock for microbial processes in recent years (Drzyzga *et al*., 2015). Besides hydrogen and some CO_2_, a major component of syngas is carbon monoxide, which can be utilized as carbon and energy source by various bacteria. These include the so‐called aerobic carboxydotrophs, such as *Oligotropha carboxidovorans* and *Alcaligenes carboxydus*, which exhibit notable physiological similarities to *R. eutropha* (Cypionka and Meyer, 1982; King, 2003). Carboxydotrophic bacteria oxidize CO with H_2_O to CO_2_ and 2 H^+^ + 2 e^−^ by a carbon monoxide dehydrogenase (CODH), thereby obtaining energy and carbon, that is usually fixed in the CBB cycle. The CODH‐associated proteins of the model organism for carboxydotrophy, *O. carboxidovorans* OM5*,* are encoded in a single 14.5‐kbp *cox* gene cluster, which is located on the organism's megaplasmid pHCG3 (Fig. S1; Fuhrmann *et al*., 2003). This cluster includes the structural genes for CODH, *coxM*,* coxS* and *coxL*, as well as genes that code for proteins mediating the post‐translational maturation of CODH (*coxD*,* coxE*,* coxF*,* coxG;* Schübel *et al*., 1995). The remaining *cox*_*Oc*_ genes, which encode transmembrane complexes (*coxB*,* coxK*,* coxI*) and a putative signal transduction system (*coxC*,* coxH*), have yet to be further characterized.

Although a utilization of carbon monoxide as a source of carbon or energy has not been shown, *R. eutropha* H16 has several characteristics, which suggest that a possible exploitation of the energy‐rich substrate CO by this bacterium is not far off. Similarly to its two hydrogenases, examined cytochromes of *R. eutropha* H16 showed low affinity towards CO (Bernard *et al*., 1974). This has led to the assumption that the respiratory system of *R. eutropha* H16 is mostly CO‐insensitive, which was supported by the study of Cypionka and Meyer (1982), who observed a substantial inhibition of growth of *R. eutropha* only at CO concentrations that exceeded those of conventional syngas by far. Furthermore, the two chromosomes of *R. eutropha* H16 harbour putative genes with significant similarities to *cox* genes of *O. carboxidovorans* (Cramm, 2009). Recently, *R. eutropha* was genetically engineered to display a cell surface‐anchoring protein consisting of CODH subunits of different anaerobic bacteria that converted CO to CO_2_ (Hyeon *et al*., 2015). However, an intracellular utilization of CO by *R. eutropha*, resulting in growth, has not been shown to this date. In this study, cultivations of *R. eutropha* H16 with a standardized artificial syngas mixture or different, defined compositions of CO, CO_2_ and H_2_ were carried out and the effect of heterologously expressed *cox* genes of *O. carboxidovorans* OM5 on growth in CO‐containing gas mixtures by *R. eutropha* was examined.

## Results and discussion

### Cultivation of strains of *R. eutropha* in the presence of carbon monoxide

**Figure 1:**
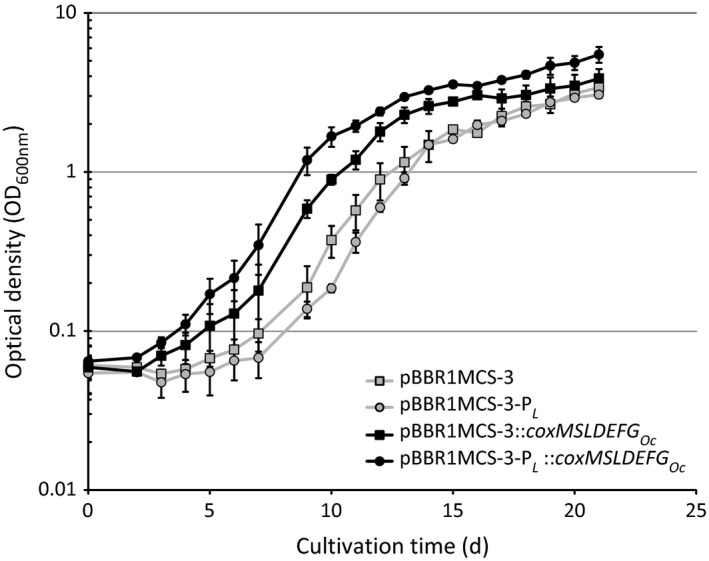
Cultivation of strains of *Ralstonia eutropha* H16, harbouring pBBR1MCS‐3 (empty vector), pBBR1MCS‐3‐P_*L*_, pBBR1MCS‐3::*coxMSLDEFG*_*Oc*_, or pBBR1MCS‐3‐P_*L*_::*coxMSLDEFG*_*Oc*_, in 50 ml mineral salts medium (Schlegel *et al*., 1961) at 30 °C and an agitation of 130 r.p.m. Cells were grown under oxic conditions in 1 L Duran flasks with an atmosphere of 30% of an artificial synthesis gas mixture (by volume 40% CO, 40% H_2_, 10% CO_2_, 10% N_2_). From day five onwards, gas atmospheres were renewed with each sampling procedure. Standard deviations of optical densities (OD_600nm_) are shown by error bars. Data were obtained from duplicate flasks of each strain over the course of two biological experiments. d, days.

To assess and enhance growth of *R. eutropha* H16 in CO‐containing atmospheres, cells of the wild type and of recombinant strains were initially cultivated aerobically with 30% (by volume) of an artificial syngas mixture. This gas mixture consisted of (by volume) 40% CO, 40% H_2,_ 10% CO_2_ and 10% N_2_, and resembled syngas compositions, which were previously used in academic studies and were actually obtained from gasification of biomass (Bridgewater, 1995; Heinrich *et al*., 2016; Revelles *et al*., 2016). To improve the utilization of syngas, of which CO is a major component, seven *cox* genes of *O. carboxidovorans* OM5 were heterologously expressed in *R. eutropha* H16. For this, *coxM*, ‐*S*, ‐*L*, ‐*D*, ‐*E*, ‐*F* and ‐*G* (locus tags: OCA5_RS17205‐260), which are the conserved *cox* genes among carboxydotrophs and are regarded as essential for carbon monoxide utilization (Santiago *et al*., 1999; King and Weber, 2007), were cloned to yield the construct pBBR1MCS‐3::*coxMSLDEFG*_*Oc*_. Furthermore, the CO_2_‐inducible promoter P_*L*_ was cloned upstream of the *cox*_*Oc*_ genes to exploit the gas exposure for enhanced expression. This promoter mediates gene transcription of the two homologous *cbb* operons of *R. eutropha* and binds the transcription activator CbbR (Jeffke *et al*., 1999). Syngas cultures of *R. eutropha* showed exponential growth after an initial lag phase of three to four days, which showed that all gluconate of the precultures had been successfully washed out from the medium prior to inoculation (Fig. 1). From day five onwards, the gas phase was renewed with every sample withdrawal to prevent a lack of carbon, or energy supply for these cultures. Recombinant *R. eutropha* strains, which harboured the *coxMSLDEFG*_*Oc*_ cluster, showed substantially enhanced growth in particular during the first 10 days of cultivation (Table 1) when compared to the control strains, which carried the plasmids pBBR1MCS‐3 or pBBR1MCS‐3‐P_*L*_. The strain harbouring pBBR1MCS‐3‐P_*L*_::*coxMSLDEFG*_*Oc*_ grew to the highest optical density (OD_600nm_: 5.48). The increased optical densities were not only a result of cell growth, as depicted by the measured cell dry weights (CDW), but also of the accumulated amounts of the carbon storage compound poly(3‐hydroxybutyrate) (poly[3HB]), which were increased by more than 20% concomitant with heterologous expression of the *cox*_*Oc*_ genes (Table 1). The crystalline short chain length polymer poly(3HB) is known to considerably affect the transmission of light through the cell material (Schlegel *et al*., 1970). Due to the technical limitation of the applied proof‐of‐concept set‐up, the exact amounts of utilized CO and CO_2_ from applied syngas were not determined. However, by means of unpaired *t*‐tests, the impact of the heterologously expressed *coxMSLDEFG*_*Oc*_ genes on growth and poly(3HB) synthesis of the respective *R. eutropha* strains (Table 1) was determined to be significant for all analysed parameters (*P* < 0.05), which suggested an increased utilization of carbon, CO and/or CO_2_, which was present in the artificial syngas mixture.

**Table 1:** Growth rates, cell dry weights and poly(3HB) accumulation of recombinant strains of *Ralstonia eutropha* H16, cultivated for 21 days. Cells were grown in 50 ml mineral salts medium (Schlegel *et al*., 1961) at 30 °C and 130 r.p.m. under oxic conditions in 1 L Duran flasks with an atmosphere of 30% of an artificial synthesis gas mixture (by volume 40% CO, 40% H_2_, 10% CO_2_, 10% N_2_). From day five onwards, gas atmospheres were renewed with each sampling procedure. Data were obtained from duplicate flasks of each strain over the course of two biological experiments. d, days; CDW, cell dry weight

*R. eutropha* pBBR1MCS‐3::	Growth rate (d^−1^; d 1–10)	Cell density (g CDW/L)	Poly(3HB) content (%, wt/wt, of CDW)
(Only vector)	0.18 ± 0.03	1.84 ± 0.18	40.8 ± 0.5
P_*L*_	0.12 ± 0.02	1.75 ± 0.13	39.0 ± 0.2
*coxMSLDEFG*_*Oc*_	0.27 ± 0.04	2.05 ± 0.23	46.4 ± 0.8
P_*L*_::*coxMSLDEFG*_*Oc*_	0.33 ± 0.03	2.62 ± 0.30	49.7 ± 0.4

**Figure 2:**
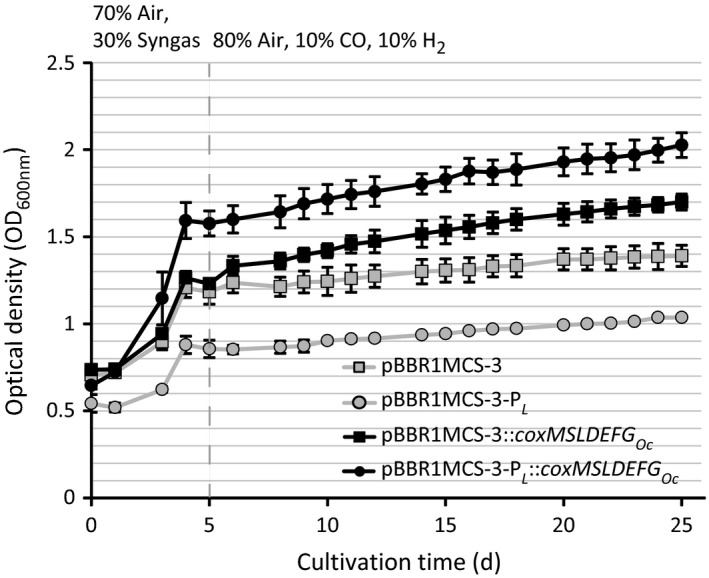
Cultivation of strains of *Ralstonia eutropha* H16, harbouring pBBR1MCS‐3 (empty vector), pBBR1MCS‐3‐P_*L*_, pBBR1MCS‐3::*coxMSLDEFG*_*Oc*_, or pBBR1MCS‐3‐P_*L*_::*coxMSLDEFG*_*Oc*_, in 1 L Duran flasks filled with 50 ml mineral salts medium (Schlegel *et al*., 1961) at 30 °C and an agitation of 130 r.p.m. Initially, cultures were grown under oxic conditions in an atmosphere of 30% of an artificial synthesis gas mixture (by volume 40% CO, 40% H_2_, 10% CO_2_, 10% N_2_). After five days of cultivation (vertical dashed line), cells were washed and transferred to fresh media. Cultures were then set to an atmosphere of (by volume) 80% air, 10% CO and 10% H_2_. Atmospheres were renewed after every five days. Standard deviations of optical densities (OD_600nm_) are represented by error bars. Data were obtained from triplicate flasks of each strain. d, days.

To verify this assumed utilization of CO, respective strains were cultivated in a similar set‐up, in which cells were cultivated with syngas for 5 days, harvested and washed with medium and then transferred to flasks containing fresh media with an aerobic atmosphere containing (i) 10% CO with 10% H_2_, (ii) 10% CO, or (iii) 10% H_2_ (by volume). To gain sufficient amounts of cell matter for poly(3HB) analysis without extending the cultivation time of the previous growth experiment (Fig. 1), the cells were initially inoculated to a higher OD_600nm_. The difference in growth during the first 5 days of cultivation between *R. eutropha* pBBR1MCS‐3::*coxMSLDEFG*_*Oc*_ and its corresponding control strain (empty vector) appeared to be less substantial than during the first days of the previous syngas cultivation (Fig. 1). Taking the higher concentrations of the inocula into account, it could be assumed that the positive effect of the vector pBBR1MCS‐3::*coxMSLDEFG*_*Oc*_ on growth with syngas was less noticeable with increasing OD_600nm_ (Fig. 1, d 10–15; Fig. 2, d 0‐5). At these higher cell densities, the comparably lower amount of CO substrate available for each individual cell, combined with the presumably low concentration of recombinant intracellular CODH_*Oc*_ resulting from basal gene expression through P_*lac*_, might have led to a relatively low rate of conversion of CO to CO_2_ for growth.

Upon the exchange of atmospheres, none of the four tested strains of *R. eutropha* was able to grow, when solely CO or H_2_ were applied (Fig. S2). In contrast, cells showed a slight, but significant increase in optical density, when 10% H_2_ was added to 10% of CO (Fig. 2). Again, *R. eutropha* pBBR1MCS‐3‐P_*L*_::*coxMSLDEFG*_*Oc*_ appeared to be the best‐performing strain and gained nearly 0.5 units of OD_600nm_ within 20 days of cultivation with CO/H_2_.

These cultivations suggested that the strains of *R. eutropha* expressing the seven *cox* genes of *O. carboxidovorans* OM5 were able to utilize carbon monoxide as carbon source but were unable to channel the resulting electrons into the respiratory chain for the generation of energy, as provision of H_2_ was essential for an increase of the OD. Surprisingly, also the control strains harbouring the respective vectors without *cox*_*Oc*_ genes showed a slight increase in OD_600nm_, however, to a much lower degree as compared to the recombinant strains. In these two‐phase cultivation experiments, the cells accumulated considerably less poly(3HB) when compared to the previous cultivations, in which syngas was applied over the entire course of the cultivation (Table 1). As syngas contained 10% of CO_2_, which in contrast to CO can be immediately assimilated through the CBB cycle, the amount of available carbon, crucial for poly(3HB) accumulation in *R. eutropha,* was likely higher when the strains were cultivated in syngas atmosphere (Anderson and Dawes, 1990). Furthermore, strains of *R. eutropha*, which harboured the *cox*_*Oc*_ genes, accumulated only slightly more poly(3HB) (*coxMSLDEFG*_*Oc*_: 18.1% ± 1.1%; P_*L*_::*coxMSLDEFG*_*Oc*_: 18.9% ± 0.9% [wt/wt of CDW]) than the corresponding control strains (empty vector: 17.0% ± 0.3%; pBBR1MCS‐3‐P_*L*_: 16.7% ± 0.5% [wt/wt of CDW]). This implied that the increased optical density of these recombinant strains was not only due to light scattering of the accumulated polymer granules, but also to cell division.

Remarkably, no growth was detected when cells were directly exposed to the aerobic CO/H_2_‐containing atmosphere after inoculating the cells from the heterotrophically grown precultures. An explanation for this might be the display of cytochrome patterns by *R. eutropha*, which evidently vary according to different growth phases and conditions (Kömen *et al*., 1991). No experiments on the cytochrome CO sensitivity of heterotrophically grown cells of *R. eutropha* H16 have been carried out; however, Probst and Schlegel (1976) reported a moderate affinity of cytochromes towards CO in CO_2_/H_2_‐cultivated cells of this bacterium. Therefore, one could speculate that cells of *R. eutropha* that were grown in syngas prior to the switch to CO/H_2_ atmosphere built a cytochrome pattern, which was more adapted to CO exposure (Fig. 2).

**Figure 3:**
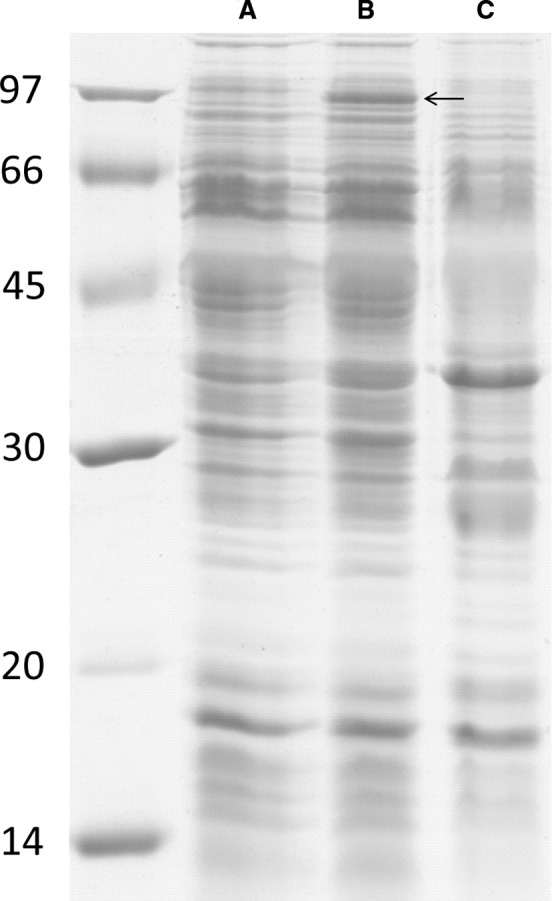
Protein patterns of the non‐soluble pellet of lysed cells of *Ralstonia eutropha*, harbouring pBBR1MCS‐3::*coxMSLDEFG*_*Oc*_ (A), pBBR1MCS‐3‐P_*L*_::*coxMSLDEFG*_*Oc*_ (B), or pBBR1MCS‐3 (empty vector; C). Cells were obtained after six days of cultivation in mineral salts medium (Schlegel *et al*., 1961) at 30 °C, an agitation of 130 r.p.m. and an atmosphere of (by volume) 70% air and 30% of an artificial syngas mixture that contained 40% CO, 40% H_2_, 10% CO_2_, 10% N_2_. Proteins of 2 mg of cell pellet were separated in an SDS polyacrylamide gel and subsequently stained with Coomassie blue. Molecular masses of proteins (in kilodaltons) are displayed on the left margin. An arrow indicates a protein band, identified as CoxL_*Oc*_ in lane ‘B’.

In both cultivation experiments, the applied P_*L*_ promoter had a positive impact on growth of the recombinant *R. eutropha* strain, which harboured the *cox*_*Oc*_ genes. This was most likely due to an increased level of expression of the episomal heterologous genes that was caused by the induced P_*L*_ promoter. Sodium dodecyl sulfate polyacrylamide gel electrophoresis (SDS PAGE) protein patterns of separated soluble supernatants of disrupted syngas‐grown cells did not show notable differences in occurrence or abundancy of proteins between the recombinant *cox*_*Oc*_ strains and the *R. eutropha* H16 wild type, harbouring the empty vector (Fig. S3). However, the protein pattern of the membrane‐containing pellet of *R. eutropha* pBBR1MCS‐3‐P_*L*_::*coxMSLDEFG*_*Oc*_ (Fig. 3) displayed a distinct spot that matched the size of the large CODH subunit CoxL_*Oc*_ (88.7 kDa; Dobbek *et al*., 1999). Applying matrix‐assisted laser desorption/ionization‐time‐of‐flight‐tandem‐mass spectrometry (MALDI‐TOF‐MS/MS) and subsequent data analysis with the Mascot search engine, this protein spot was clearly identified as CoxL_*Oc*_ (Protein score, 105.5; Confidence interval [C.I.%], 100%). Other Cox subunits or accessory proteins could not be detected; this may be due to (i) an overlay of proteins, (ii) weakspecific binding of the Coomassie dye or (iii) partial 3′‐terminal degradation of mRNA of a respective gene, downstream of *coxL*_*Oc*_. Moreover, the expression of *cox*_*Oc*_ genes under control of the pBBR1MCS‐3 standard P_*lac*_ promoter, which cannot be induced but exhibits basal activity in *R. eutropha* strains, presumably occurred at a low level. This led to protein concentrations that cannot be detected in SDS PAGE protein patterns.

The detection of at least the CoxL_*Oc*_ subunit in disrupted cells of *R. eutropha* pBBR1MCS‐3‐P_*L*_::*coxMSLDEFG*_*Oc*_ together with the increased growth in CO‐containing atmospheres of this strain indicated that the applied P_*L*_ is a suitable promoter for heterologous gene expression in syngas cultivations with *R. eutropha*. The CO_2_, which was responsible for induction of the P_*L*_ promoter, was solved in the culture broth and additionally emerged from the assumed intracellular conversion of CO to CO_2_. In contrast, the presence of the P_*L*_ promoter on the control plasmid pBBR1MCS‐3‐P_*L*_ leads to decreased growth (Figs 1 and 2). As episomal copies of P_*L*_ could capture the regulating CbbR proteins, which are required for transcription of the *cbb* genes of *Ralstonia eutropha*, CO_2_ fixation of the respective strain might have been hampered (Bowien and Kusian, 2002).

As an additional observation, the positive impact of the *cox*_*Oc*_ genes on growth of the recombinant *R. eutropha* strains in CO‐containing atmospheres was more substantial in syngas atmosphere (Fig. 1) than in the two‐phase cultivation, where CO was the sole carbon source (Fig. 2). Possibly, the presence of CO_2_, which is a natural substrate of *R. eutropha*, leads to an increased fitness of the recombinant strains as compared to cultivations, where cells solely relied on carbon monoxide. As a mere hypothesis, the recombinant CODH_*Oc*_ could not only provide limited amounts of CO_2_ as carbon source for *R. eutropha*, but as a side‐effect could also dispose the medium of accumulating CO, which would be partially inhibiting growth at otherwise increasingly higher concentrations (Cypionka and Meyer, 1982). Despite their inability to gain sufficient amounts of energy from carbon monoxide, this possible detoxification effect could explain the enhanced productivity of the recombinant *R. eutropha cox*_*Oc*_ strains in the conducted syngas cultivations, as compared to the wild type.

### *In vitro* analysis of carbon monoxide oxidation by strains of *R. eutropha* H16

To demonstrate the ability of the recombinant *R. eutropha* strains to oxidize CO to CO_2_ *in vitro*, an altered protocol of the photometric assay of Meyer and Schlegel (1978), in which the oxidation of CO is coupled to the reduction of methylene blue, was applied. For this, duplicate flasks with cells of the different generated strains of *R. eutropha* were cultivated in 30% of syngas (by volume) to ODs_600nm_ of 0.8–1.0. The wild type of *O. carboxidovorans* OM5, which was cultivated under oxic conditions with (by volume) 30% CO for 10 days to an OD_600nm_ of 1.0, was used as a reference. Following the disruption of cells, the soluble supernatant and the non‐soluble pelleted fraction were analysed for CODH activity. Of the tested *cox*_*Oc*_ *‐* and control strains of *R. eutropha* H16, cell extracts of the two strains, which expressed the *coxMSLDEFG*_*Oc*_ cluster, showed an oxidation of carbon monoxide in the photometric enzyme assay. However, CODH activity of the applied recombinant *R. eutropha* cell extract was found exclusively in the pellet, containing cytoplasmic membranes of lysed cells. This was in accordance with the detection of CoxL_*Oc*_, in protein patterns, prepared from the non‐soluble cell pellet of *R. eutropha* pBBR1MCS‐3‐P_*L*_::*coxMSLDEFG*_*Oc*_ (Fig. 3). Due to the constitutive episomal expression of *coxG*_*Oc*_, which codes for the CODH membrane‐anchoring protein, the recombinant CODH might be attached to the cytoplasmic membrane of *R. eutropha* during all stages of growth. In contrast, the native CO dehydrogenases of *O. carboxidovorans* OM5 are attached to the cytoplasmic membrane and diffused in the cytoplasm at different ratios, depending on the respective growth phase (Rohde *et al*., 1984). Consequently, the soluble cell fraction of *O. carboxidovorans* OM5 catalysed the oxidation of 28.5 nanomoles CO·min^−1^·mg protein^−1^, whereas the pellet of lysed cells oxidized CO at a rate of 4.8 nanomoles·min^−1^·mg dried cell pellet^−1^. The activity of CO oxidation of the *R. eutropha cox*_*Oc*_ strains appeared to be clearly lower than for the reference strain *O. carboxidovorans* OM5, as pellets of lysed *R. eutropha* cells harbouring the plasmids pBBR1MCS‐3::*coxMSLDEFG*_*Oc*_ and pBBR1MCS‐3‐P_*L*_::*coxMSLDEFG*_*Oc*_ oxidized CO at rates of 0.7 and 0.9 nanomoles·min^−1^·mg dried cell pellet^‐1^. The recorded increase in the rate of CO oxidation resulting from application of the P_*L*_ promoter was approximately 23%, although the cellular abundance of at least CoxL_*Oc*_, which appeared to be much higher for this strain (Fig. 3), would have suggested an even more increased activity of respective cell extract. A potential approach to further increase in CO oxidation by the recombinant strains could be the further fine tuning of their *cox*_*Oc*_ gene expression, aiming at the equal abundance of all CODH_*Oc*_ subunits, which could result in an increased amount of functional cellular CODH enzymes.

### Cultivation of strains of *R. eutropha* H16 with ^13^CO

To demonstrate that the *R. eutropha cox*_*Oc*_ strains were not only able to oxidize carbon monoxide but also to assimilate the CO‐derived carbon, the strains were cultivated with ^13^C‐labelled carbon monoxide as the sole C source. For this purpose, a shortened two‐stage cultivation was carried out, which comprised 5 days of growth with the artificial syngas mixture followed by 10 days of exposure to (by volume) 10% ^13^CO + 10% H_2_ (Fig. S4). Along with the cell numbers of withdrawn samples, conversion of ^13^CO into poly(3HB) was determined by gas chromatography–mass spectrometry (GC‐MS), as poly(3HB) derives from the central metabolite acetyl coenzyme A (acetyl‐CoA). Therefore, ^13^C enrichment in the characteristic *m*/*z* 103 fragment of methanolyzed poly(3HB) was determined (Table 2). The recombinant *cox*_*Oc*_ strains of *R. eutropha* H16 again showed only a slight increase in optical density, but incorporated substantial amounts of ^13^C‐labelled carbon, which derived from the applied ^13^CO, into the backbone of the accumulated poly(3HB) after 10 days of cultivation with ^13^CO (Table 2). Taking the natural abundance of carbon, oxygen and hydrogen isotopes into account, most of these labelled monomers appeared to contain three ^13^C atoms *(m*/*z*: 106), whereas only a small amount of monomers containing one ^13^C atom (*m*/*z*: 104) and no monomers containing two ^13^C (*m*/*z* 105) atoms were detected (Fig. S5). As cultures were supplied with exclusively ^12^C containing syngas (d 0–5) or ^13^CO (d 5–15), the occurrence of polymer constituents containing both ^12^C and ^13^C atoms could have resulted from ^12^C which was mobilized from the central metabolism, biomass or poly(3HB) into poly(3HB) precursors during growth with ^13^CO. The amount of incorporated ^13^C was higher than one could expect from the small increase in accumulated poly(3HB) during cultivation with ^13^CO/H_2_ (Table 2). This may be due to the simultaneous synthesis and degradation of poly(3‐hydroxyalkanoate) by *R. eutropha* H16, first reported by Doi *et al*. (1990). The 3HB constituents originating from non‐labelled carbon compounds of syngas were thereby steadily replaced by constituents derived from ^13^CO. Furthermore, the cell number of the strains, harbouring *cox*_*Oc*_ genes slightly increased over the course of the exposure to ^13^CO and H_2_ (Table 2), which suggested that carbon from CO was utilized for cell growth. A minor increase in cell number was also recorded for the *R. eutropha* control strains harbouring the empty pBBR1MCS‐3(‐P_*L*_), which, however, did not meet statistical significance (*P* > 0.05). The pH of the cultures after 15 days of cultivation with ^13^CO/H_2_ was in the range of 6.6 (± 0.2), whereas cultures displayed an approximate pH of 6.7 (± 0.1) after the five‐day syngas cultivation phase, which implied that this parameter did not affect growth with CO and H_2_. These results, combined with the foregoing growth experiments and the photometric enzyme assay, provided unequivocal evidence that the recombinant *R. eutropha* strains generated in this study were able to oxidize and utilize CO as the sole carbon source by CODH_*Oc*_‐mediated conversion to CO_2_.

**Table 2:** Changes in optical density, cell number and poly(3HB) accumulation as well as incorporation of ^13^C into poly(3HB) of generated strains of *Ralstonia eutropha* H16 after ten days of cultivation with 80% air, 10% ^13^CO and 10% H_2_ (by volume). Cells were initially grown in 100 ml mineral salts medium (Schlegel *et al*., 1961) at 30 °C and 130 r.p.m. under oxic conditions in 1 L Duran flasks with an atmosphere of 30% of an artificial synthesis gas mixture (by volume 40% CO, 40% H_2_, 10% CO_2_, 10% N_2_). After five days of cultivation, 50 ml of culture broth was harvested, and the remaining 50 ml was washed and transferred to fresh media. Cultures were then set to an atmosphere of 80% air, 10% ^13^CO and 10% H_2_ (by volume), which was renewed after five further days of cultivation, before the remaining cultures were harvested. Data were obtained from duplicate flasks. M0 to M3 display the m + 0 to m + 3 enrichments of the 3HB‐methyl ester′s *m*/*z* 103 fragment with ^13^C, detected by GC‐MS as described by Tan *et al*. (2016). The fractions of ^13^C‐enriched 3HB constituents were determined after correcting for natural isotope abundances

*R. eutropha* pBBR1MCS‐3::	Δ OD_600nm_	Δ Cell number (10^8^ ml^−1^)	Δ poly(3HB) (wt/wt of CDW)	Incorporation of ^13^C (M0/M1/M2/M3)
(Only vector)	+0.08 ±0.02	+0.21 ±0.11	+0.2% ±0.1%	99.8/0.2/–/– ±0.2/0.2/–/–
P_*L*_	+0.06 ±0.00	+0.09 ±0.05	−0.1% ±0.1%	100/–/–/– ±0.0/–/–/–
*coxMSLDEFG*_*Oc*_	+0.22 ±0.00	+0.95 ±0.13	+1.6% ±0.4%	92.6/0.8/–/6.6 ±0.6/0.2/–/0.4
P_*L*_::*coxMSLDEFG*_*Oc*_	+0.23 ±0.04	+1.04 ±0.19	+2.0% ±0.5%	88.6/2.2/–/9.2 ±0.8/1.0/–/0.2

To this point, the cause for the inability to sufficiently exploit the reducing power of CO for cell growth remains unclear but cannot be experimentally solved within this study. A critical point when discussing the transfer of electrons from the CODH_*Oc*_ to the respiratory chain is the identification of the responsible electron acceptor. Although cytochrome *b*_561_ was widely considered to accept the reducing equivalents from the CODH of *O. carboxidovorans* (Cypionka and Meyer, 1983), recent experiments suggested that the common redox carrier ubiquinone (coenzyme Q10) initially interacts with the CODH flavin site (Wilcoxen *et al*., 2011). Furthermore, sequence analysis revealed that *R. eutropha* H16 possesses a cytochrome *b* large subunit (HoxZ) with a high identity to cytochrome *b*_561_ of *O. carboxidovorans* OM5 (BLASTP expect value, 4e^−83^; identities, 147/242 [60%]; positives, 185/242 [76%]; gaps, 3/242 [1%]; Altschul *et al*., 1997). Therefore, *R. eutropha* could potentially transfer reducing equivalents from CO to its respiratory chain either way. Possibly, an imperfect insertion of the recombinant CODH into the cytoplasmic membrane sterically hindered the transmission of reducing equivalents to their acceptor in the *R. eutropha cox*_*Oc*_ strains. In this case, the fate of the electrons of the reduced CODH would be unclear at this point. A simpler explanation would be that the amount of CO, which is oxidized by the recombinant CODH, is insufficient to feed the energy demand of the cell, for instance, CO_2_ fixation through the CBB cycle. This was implied by the considerably lower CO oxidation rates of the recombinant *R. eutropha* strains as compared to the reference *O. carboxidovorans* OM5. Several bacteria, e.g. of the genus *Mesorhizobium*, can oxidize CO but are unable to utilize the resulting CO_2_ for cell growth, due to the absence of CO_2_‐fixing mechanisms and are thus referred to as ‘carboxydovores’ (King, 2003). In contrast to these lithoheterotrophic ‘carboxydovores’, the recombinant strains of *R. eutropha* cultivated in this study metabolized CO as source of carbon, but both were dependent on molecular hydrogen for cell growth. Consequently, the CO‐related lifestyle of the engineered strains of *R. eutropha* could be referred to as ‘carboxyhydrogenotrophic’.

No *in vitro* CO oxidation was detected for the *R. eutropha* control strains harbouring the empty pBBR1MCS‐3 or pBBR1MCS‐3‐P_*L*_, which consequently were therefore either unable to oxidize CO under the applied conditions, or their reactions were below the detection level respectively. Previous studies by King and Weber (2007), as well as Cramm (2009) reported the presence of multiple *coxL* homologues in the genome of *R. eutropha* H16. Of these, one putative *coxL* gene (locus tag: H16_RS02155) is located in a *coxSLMDEGI* cluster, which was shown to be constitutively transcribed, at least under heterotrophic growth conditions, in foregoing studies by Peplinski *et al*. (2010). This cluster was annotated to putatively encode a xanthine oxidase, an enzyme that has diverse substrate specificities and is phylogenetically close to CODHs of, e.g. *O. carboxidovorans* (King, 2003; Hille, 2005). Still, a possible activity of this putatively formed enzyme towards carbon monoxide remains to be investigated in further studies.

Due to its metabolic versatility, *R. eutropha* H16 has been exploited to synthesize biobased products from various heterotrophic and autotrophic carbon sources (Volodina *et al*., 2016). Yet CO has been widely ignored as substrate for this bacterium, despite the importance of utilizing all carbon compounds of waste gases for a desirable circular economy. In this study, the utilization of CO as part of the economically and ecologically sustainable feedstock syngas by *R. eutropha* H16 through heterologous expression of the *coxMSLDEFG* genes of *O. carboxidovorans* OM5 has been demonstrated both *in vivo* and *in vitro*. A conceivable benefit of this CODH_*Oc*_‐mediated oxidation of CO over the recently developed cell surface‐attached CODH (Hyeon *et al*., 2015) could be the intracellularly nascent CO_2_, that is readily available to be incorporated into the CBB cycle by the ribulose 1,5 bisphosphate carboxylase. In contrast, extracellular CO_2_ enters the cell as HCO_3_^−^ and thus initially must be converted by the *R. eutropha* carbonic anhydrase Cag (Gai *et al*., 2014). By oxidation of toxic CO to CO_2_ and the resulting supply of additional accessible carbon, potential syngas processes involving *R. eutropha* H16, could exhibit enhanced productivity and efficiency, as shown for the commercially applicable biopolymer poly(3HB) in this study.

## Experimental procedures

### Microorganisms, plasmids and oligonucleotides

Bacterial strains, plasmids and oligonucleotides, which were used in this study, are listed in Table S1. *Escherichia coli* TOP10 was used for cloning procedures that included the propagation and isolation of generated plasmids. *E. coli* C41 was used as a negative reference strain in the photometric CODH assay.

### Cultivation of bacteria

*Escherichia coli* strains were grown in 5 ml of lysogeny broth (Sambrook *et al*., 1989) in test tubes at 37 °C and an agitation speed of 130 r.p.m. *O. carboxidovorans* OM5 and strains of *R. eutropha* H16 were grown at 30 °C in mineral salts medium (Schlegel *et al*., 1961), which contained (per litre) 4.5 g Na_2_HPO_4_·2 H_2_O, 1.5 g KH_2_PO_4_, 1.0 g NH_4_Cl, 0.2 g MgSO_4_·7 H_2_O, 0.02 g CaCl_2_, 1.2 mg NH_4_Fe(III) citrate and 10 μl of 10 000‐fold SL6 (Pfennig, 1974). For growth on solid media and for precultures, 1% (wt/vol) of sodium gluconate for *R. eutropha* or 0.3% (wt/vol) of sodium acetate for *O. carboxidovorans* OM5 was added to the medium. For precultures, cells were grown in 20 ml of medium in 100 ml Erlenmeyer flasks and an agitation of 130 r.p.m. for 24–32 h. Cells were washed with carbon‐free mineral salts medium by centrifugation for 15 min at 4000 *g* before inoculating the main culture with 1 ml of a concentrated cell suspension. Cultivations of cells of *R. eutropha* with different gas mixtures were carried out at a volume of 50 ml in baffled 1 L Duran flasks, which were sealed with butyl rubber plugs. Upon evacuation of the respective defined amount of air, flasks were filled with an artificial syngas mixture (by volume, 40% CO, 40% H_2_, 10% CO_2_ and 10% N_2_; Air Liquide, Bottrop, D) to a final concentration of 30%. Similarly, atmospheres of (by volume) 10% ^(13)^CO, 10% H_2_ and 10% CO_2_ (Air Liquide, Bottrop D/Eurisotop, Saarbrücken, D) or different combinations of these gas concentrations were established. Autotrophic cultivations of *O. carboxidovorans* OM5 were carried out in an atmosphere of (by volume) 70% air and 30% CO. Optical densities of samples were determined at 600 nm (OD_600nm_). The cell count (·ml^−1^) was determined from samples, diluted between 1:10 and 1:50, using a Thoma counting chamber. Cell harvest was carried out by centrifugation (15 min, 4000 *g*, 4 °C). Cultures of recombinant strains contained tetracycline at concentrations of 25 μg ml^−1^ for *R. eutropha* and 12.5 μg ml^−1^ for *E. coli*. During gas cultivations, tetracycline was added repeatedly after 7 days of cultivation with recombinant strains of *R. eutropha* to maintain plasmid stability.

### Construction of vectors and generation of recombinant strains of *E. coli* and *R. eutropha*

Nucleic acids were processed according to Sambrook *et al*. (1989). DNA fragments were amplified from genomic DNA of *O. carboxidovorans* OM5 and *R. eutropha* H16 by applying the Phusion High‐Fidelity DNA Polymerase (New England Biolabs, Ipswich, MA, USA) with oligonucleotides, listed in Table S1. FastDigest restriction enzymes (Thermo Scientific, Waltham, MA, USA) were used to digest DNA fragments, before ligation into a likewise digested target plasmid applying T4 DNA ligase (Thermo Scientific, Waltham, MA, USA). To propagate, isolate and validate generated hybrid plasmids, chemically competent cells of *E. coli* TOP10 were transformed, applying the method of Hanahan (1983). Re‐isolated plasmids were then transferred into *R. eutropha* H16 by electroporation (Aneja *et al*., 2009). The vector pBBR1MCS‐3::*coxMSLDEFG*_*Oc*_ was generated by digesting the amplified *coxMSLDEFG*_*Oc*_ DNA fragment with SpeI and SacI, and subsequent ligation into a likewise digested pBBR1MCS‐3 vector. SpeI‐digested P_*L*_ fragments were then inserted into the corresponding site to yield the vector pBBR1MCS‐3‐P_*L*_::*coxMSLDEFG*_*Oc*_.

### Preparation of cell extract and analysis of protein patterns

Harvested cells were washed and resuspended in 50‐mm KH_2_PO_4_‐KOH buffer (pH 7.0) with added protease inhibitor (cOmplete ULTRA Tablets, Roche, Basel, CH), and cell extracts of *R. eutropha* were obtained by sonication and subsequent centrifugation (10 min, 13 000 *g*, 4 °C). Protein concentrations were determined employing the method of Bradford (1976), and 40 μg of protein were heated in denaturing buffer for 10 min at 95 °C. Upon separation by SDS PAGE (Laemmli, 1970), proteins were stained with Coomassie brilliant blue R‐250. Proteins were identified by MALDI‐TOF‐MS/MS. For this, respective protein spots were excised from SDS gels and transferred to 1.5 ml reaction tubes containing 50 μl 10% (vol/vol) acetic acid. Protein samples were subjected to MALDI‐TOF‐MS/MS analysis as described by Wolf *et al*. (2008). Applying a 5800 Proteomics Analyzer (AB Sciex, Framingham, MA, USA), the spectra were recorded in a reflector mode in a mass range from 900 to 3700 Da with a focus mass of 200 Da. Acquired MS data were compared to the proteome database of *O*. *carboxidovorans* OM5 and *R. eutropha* H16 using the Mascot engine (version 2.1.0.4).

### Determination of CODH activity in cell extracts

CODH activity in cell lysates was determined with a method modified according to Meyer and Schlegel (1978). In the present protocol, the oxidation of glucose by a glucose oxidase/catalase mix to dispose residual oxygen was omitted, as it affected the reduction of methylene blue from CO in the applied experimental set‐up. The reaction was started by adding 20 μl of soluble cell extract (2–8 mg ml^−1^ of protein) or 1–3 mg of resuspended wet cell pellet to 1.8 ml of the CO–saturated 50 mm methylene blue solution.

### Analysis of poly(3HB) content of cells

Upon cell harvest, cells were lyophilized, and 5–10 mg of each sample was subjected to acidic methanolysis as described by Brandl *et al*. (1988). Synthesized 3HB‐methyl esters were then quantified by gas chromatography (Timm *et al*., 1990), using an Agilent 6850 gas chromatograph (GC), which was equipped with a BP21 polyethylene glycol capillary column (50 m by 0.22 mm; 250 nm film thickness) and a flame ionization detector (Agilent Technologies, Waldbronn, D). To determine the content of ^13^C, obtained 3HB‐methylesters were analysed by GC‐MS according to Tan *et al*. (2016). Applying an Agilent 6890 GC, which was connected to an Agilent HP 5973 mass spectrometer (MS), the samples were separated on a BPX35 polyethylene glycol capillary column (60 m by 0.22 mm; 250 nm film thickness; SGE Deutschland GmbH, Darmstadt, D). For this, the temperature programme described by Andreeßen *et al*. (2010) was applied.

## Conflict of interest

The authors have no conflict of interest to declare.

## Supporting information


**Table S1.** Microorganisms, target vectors and oligonucleotides, which were applied in this study.
**Fig. S1.** Cluster of *cox*genes in *Oligotropha carboxidovorans* OM5.
**Fig. S2.** Cultivation of strains of *R. eutropha* H16, harbouring pBBR1MCS‐3 (empty vector), pBBR1MCS‐3‐P_*L*_, pBBR1MCS‐3::*coxMSLDEFG*_*Oc*_ or pBBR1MCS‐3‐P_*L*_::*coxMSLDEFG*_*Oc*_ in 1L Duran flasks filled with 50 ml mineral salts medium (Schlegel *et al*., 1961) at 30 °C and 130 r.p.m.
**Fig. S3.** Protein patterns of the soluble supernatant of lysed cells of *R. eutropha*, harbouring pBBR1MCS‐3::*coxMSLDEFG*_*Oc*_ (A), pBBR1MCS‐3‐P_*L*_::*coxMSLDEFG*_*Oc*_ (B); or pBBR1MCS‐3 (empty vector; C).
**Fig. S4.** Cultivation of strains of *R. eutropha* H16, harbouring pBBR1MCS‐3 (empty vector), pBBR1MCS‐3‐P_*L*_, pBBR1MCS‐3::*coxMSLDEFG*_*Oc*_, or pBBR1MCS‐3‐P_*L*_::*coxMSLDEFG*_*Oc*_, in 1L Duran flasks at 30 °C and an agitation of 130 r.p.m.
**Fig. S5.** Mass spectra of 3‐hydroxybutyrate methyl esters, extracted from cells of *Ralstonia eutropha* pBBR1MCS‐3‐P_*L*_::*coxMSLDEFG*_*Oc*_.
