# Supplementary material for: Studies on the aerobic utilization of synthesis gas (syngas) by wild type and recombinant strains of Ralstonia eutropha H16
Source: Microb Biotechnol. 2017 Oct 13;11(4):647–56. doi: 10.1111/1751-7915.12873 (PMC6011924; doi:10.1111/1751-7915.12873)
Supplement: Supplementary file 1 — Table S1. Microorganisms, target vectors and oligonucleotides, which were applied in this study. Fig. S1. Cluster of coxgenes in Oligotropha carboxidovorans OM5. Fig. S2. Cultivation of strains of R. eutropha H16, harbouring pBBR1MCS‐3 (empty vector), pBBR1MCS‐3‐PL, pBBR1MCS‐3::coxMSLDEFG Oc or pBBR1MCS‐3‐PL::coxMSLDEFG Oc in 1L Duran flasks filled with 50 ml mineral salts medium (Schlegel et al., 1961) at 30 °C and 130 r.p.m. Fig. S3. Protein patterns of the soluble supernatant of lysed cells of R. eutropha, harbouring pBBR1MCS‐3::coxMSLDEFG Oc (A), pBBR1MCS‐3‐PL::coxMSLDEFG Oc (B); or pBBR1MCS‐3 (empty vector; C). Fig. S4. Cultivation of strains of R. eutropha H16, harbouring pBBR1MCS‐3 (empty vector), pBBR1MCS‐3‐PL, pBBR1MCS‐3::coxMSLDEFG Oc, or pBBR1MCS‐3‐PL::coxMSLDEFG Oc, in 1L Duran flasks at 30 °C and an agitation of 130 r.p.m. Fig. S5. Mass spectra of 3‐hydroxybutyrate methyl esters, extracted from cells of Ralstonia eutropha pBBR1MCS‐3‐PL::coxMSLDEFG Oc. [file MBT2-11-647-s001.docx]

Supporting Information

Table S1: Microorganisms, target vectors and oligonucleotides, which were applied in this study. Genomic DNA of *R. eutropha* H16 or *O. carboxidovorans* OM5 served as templates for amplification of DNA fragments. Restriction sites of oligonucleotides, which were used for cloning of the respective DNA fragments, are highlighted in bold type.

| Organisms, plasmids, or oligonucleotides | Relevant characteristics or sequence (5‘🡪3‘) | Source or reference |
| --- | --- | --- |
| *E. coli* C41 | F *ompT hsdSB* (*rB^-^ mB^-^) gal dcm* (DE3) | Lucigen (Middleton, WI, USA) |
| *E. coli* TOP10 | F^-^ *mcrA* *Δ*(*mrr-hsdRMS-mcrBC*) *ϕ80lacZΔM15* Δ*lacX74* *nupG recA1* *araD139* Δ*(ara-leu)7697 galE15 galK16 rpsL(StrR) endA1 λ*^-^ | Life Technologies (Darmstadt, D) |
| *R. eutropha* H16 | Wild type | Wilde, 1962 |
| *O. carboxidovorans* OM5 | Wild type | Meyer and Schlegel, 1979 |
|  |  |  |
| pBBR1MCS-3 | Tc^r^ | Kovach *et al.*, 1995 |
| pBBR1MCS-3-P*_L_* | Tc^r^, P*_L_* | This study |
| pBBR1MCS-3::*coxMSLDEFG_Oc_* | Tc^r^, *O. carboxidovorans* *coxM, coxS, coxL, coxD, coxE, coxF, coxG* | This study |
| pBBR1MCS-3-P*_L_*::*coxMSLDEFG_Oc_* | Tc^r^, P*_L_*, *O. carboxidovorans* *coxM, coxS, coxL, coxD, coxE, coxF, coxG* | This study |
|  |  |  |
| coxMoc_fwd_SpeI | GG**ACTAGT**AGGAGGGTCGCCGTGATACCTGGTTC | This study |
| coxGoc_rev_SacI | CG**GAGCTC**TCAAACTCGCGACGAGCAG | This study |
| PL_fwd_SpeI | GT**ACTAGT**CCACGGTGACGAAGATCTG | This study |
| PL_rev_SpeI | CT**ACTAGT**TGATGCGGAACAGCGCCAG | This study |





**Fig. S1:** Cluster of *cox*-genes in *Oligotropha carboxidovorans* OM5. Open reading frames (ORFs) of the CODH-structural genes *coxM*, -*S*, and –*L*, are highlighted in dark grey. Genes of proteins that are essential for maturation (*coxD, -E,* and -*F*) and membrane-implementation of the CODH (*coxG*) are marked with a light grey background. Due to their unknown or non-essential function, unmarked ORFs were excluded from heterologous expression. kbp, kilobase pairs.





Fig. S2: Cultivation of strains of *R. eutropha* H16, harbouring pBBR1MCS-3 (empty vector), pBBR1MCS-3-P*_L_*, pBBR1MCS-3::*coxMSLDEFG_Oc_* or pBBR1MCS-3-P*_L_*::*coxMSLDEFG_Oc_* in 1‑L Duran flasks filled with 50 mL mineral salts medium (Schlegel *et al.*, 1961) at 30 °C and 130 rpm. Initially all strains were grown under oxic conditions in an atmosphere of 30% of artificial synthesis gas (by volume 40% CO, 40% H_2_, 10% CO_2_, 10% N_2_). After five days of cultivation (vertical dashed line), cells were washed and transferred to fresh media. Cultures were then set to an atmosphere of 90% air and 10% CO (broken lines, empty symbols) or 90% air and 10% H_2_ (solid lines, filled symbols). Atmospheres were then renewed after every 4-5 days. Optical densities of samples were determined at 600 nm. Data were obtained from duplicate flasks. d, days.

**
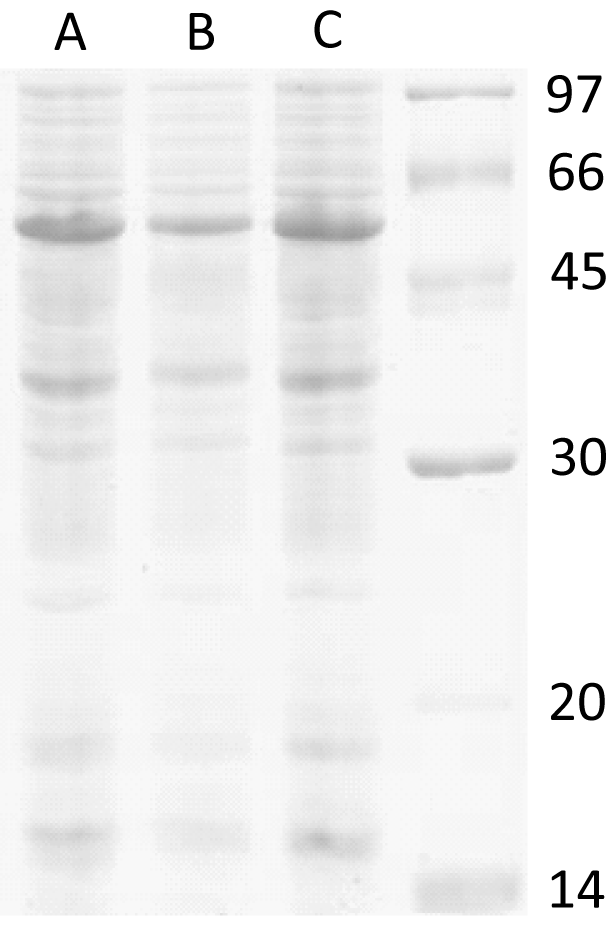
**

**Fig. S3:** Protein patterns of the soluble supernatant of lysed cells of *R. eutropha*, harbouring pBBR1MCS-3::*coxMSLDEFG_Oc_* (A), pBBR1MCS-3-P*_L_*::*coxMSLDEFG_Oc_* (B); or pBBR1MCS-3 (empty vector; C). Cells were obtained after six days of cultivation in 1‑L Duran flasks filled with 50 mL mineral salts medium (Schlegel *et al.*, 1961) at 30 °C, an agitation of 130 rpm and an atmosphere of (by volume) 70% air plus 30% of an artificial syngas mixture that contained 40% CO, 40% H_2’_, 10% CO_2_, 10% N_2_. A quantity of 40 µg of protein was separated in an SDS polyacrylamide gel and subsequently stained with Coomassie blue. Molecular masses of proteins (in kilodaltons) are displayed on the right.





Fig. S4: Cultivation of strains of *R. eutropha* H16, harbouring pBBR1MCS-3 (empty vector), pBBR1MCS-3-P*_L_*, pBBR1MCS-3::*coxMSLDEFG_Oc_*, or pBBR1MCS-3-P*_L_*::*coxMSLDEFG_Oc_*, in 1‑L Duran flasks at 30 °C and an agitation of 130 rpm. Cells were initially grown in 100 mL mineral salts medium (Schlegel *et al.*, 1961) at 30 °C and 130 rpm under oxic conditions with an atmosphere of 30% of an artificial synthesis gas mixture (by volume 40% CO, 40% H_2_, 10% CO_2_, 10% N_2_). After five days of cultivation (vertical dashed line), 50 mL of culture broth were harvested and the remaining 50 mL were washed and transferred to fresh media. Cultures were then set to an atmosphere of (by volume) 80% air, 10% ^13^CO and 10% H_2_, which was renewed after five further days of cultivation, before the remaining cultures were harvested. Optical densities (solid lines, large symbols) and cell numbers (broken lines, small symbols) were obtained from duplicate flasks. Standard deviations are shown by error bars.


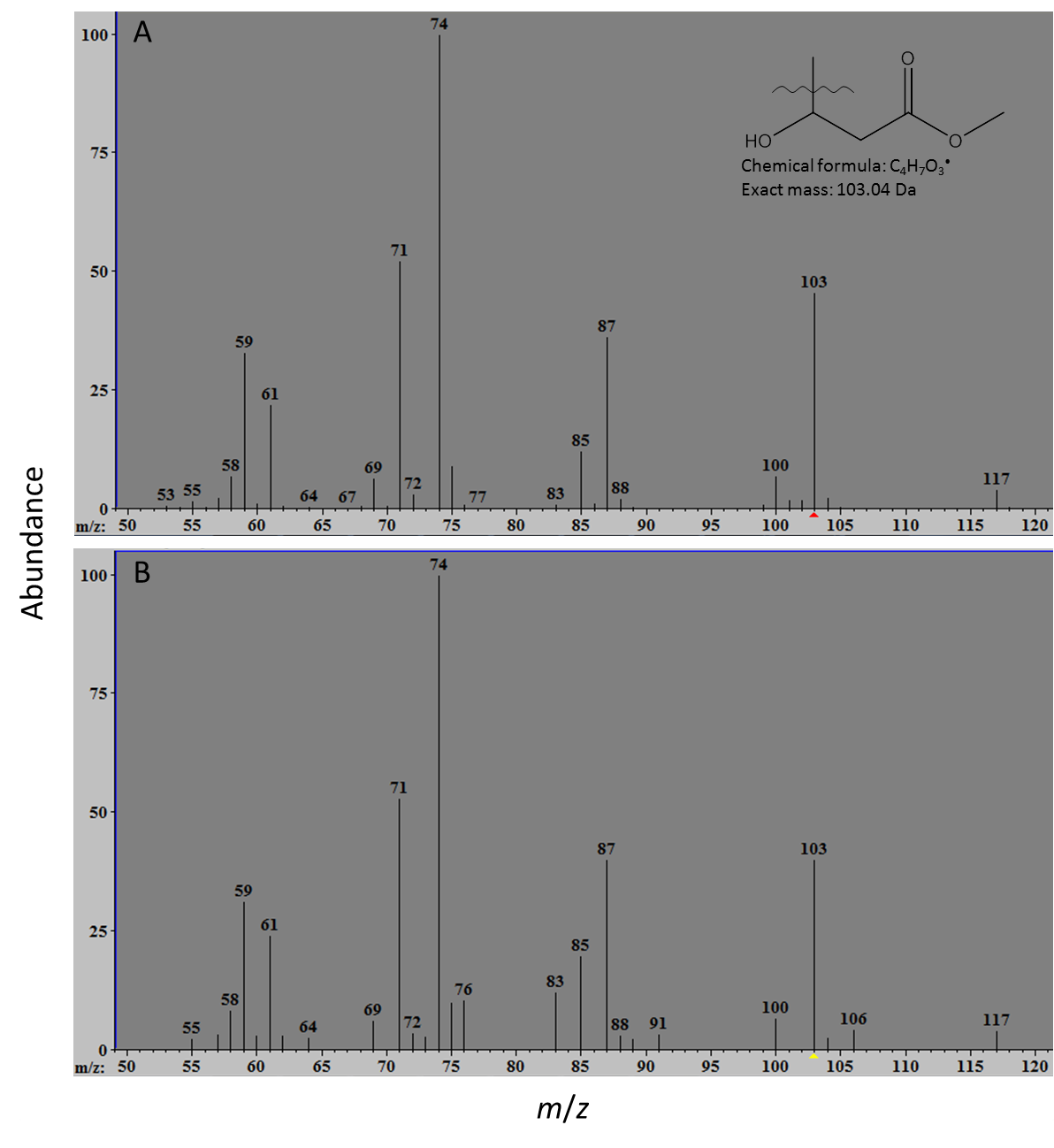
Fig. S5: Mass spectra of 3-hydroxybutyrate methyl esters, extracted from cells of *Ralstonia eutropha* pBBR1MCS-3-P*_L_*::*coxMSLDEFG_Oc_*. A: Cells cultivated aerobically for five days with 30% of artificial synthesis gas mixture (by volume 40% CO, 40% H_2_, 10% CO_2_, 10% N_2_). B: Cells cultivated aerobically for ten days with (by volume) 10% ^13^CO and 10% H_2_. The inset on the upper right shows the characteristic *m*/*z* 103 fragment, analyzed for isotope enrichment according to Tan *et al.* (2016).

**References**

Schlegel, H. G., Kaltwasser, H., Gottschalk, G. (1961) Ein Submersverfahren zur Kultur Wasserstoff oxydierender Bakterien: Wachstumsphysiologische Untersuchungen. *Arch Mikrobiol* **38**: 209-222.

Tan, G.Y.A., Ge, L., Pan, C., Tan, S.N., Wang, J.Y. (2016) Current and emerging advanced technologies for biopolyesters characterization, p 303-402. *In* Koller, M. (ed.) Recent Advances in Biotechnology, vol 2. Bentham Science Publishers, Sharjah, UAE.
